# Integrating automated electromagnetic tracking-based needle reconstruction in the intraoperative high-dose-rate prostate brachytherapy workflow

**DOI:** 10.1016/j.phro.2025.100884

**Published:** 2025-12-03

**Authors:** Ioannis Androulakis, Jérémy Godart, Miranda E.M.C. Christianen, Henrike Westerveld, Lorne Luthart, Remi A. Nout, Mischa S. Hoogeman, Inger-Karine K. Kolkman-Deurloo

**Affiliations:** a Department of Radiotherapy, Erasmus MC Cancer Institute, University Medical Center, Rotterdam, the Netherlands; b Department of Medical Physics and Informatics, HollandPTC, Delft, the Netherlands

**Keywords:** Electromagnetic tracking, Brachytherapy, Prostate, Treatment planning

## Abstract

**Background and Purpose:**

Manual transrectal ultrasound (TRUS) reconstruction, currently used in intraoperative prostate brachytherapy, is error‑prone. Automated implant reconstruction with electromagnetic tracking (EMT) has the potential to improve accuracy. The aim of this study was to implement and validate afterloader‑integrated EMT for automated implant reconstruction.

**Materials and Methods:**

An afterloader prototype equipped with an integrated EMT system was used in conjunction with a TRUS-based treatment planning system and setup. A workflow was developed to transform the EMT measurements into the implant reconstruction and import this into the treatment planning software. The workflow was retrospectively evaluated using clinical data from 14 patients. To align the EMT measurements with the treatment-planning coordinate system without a TRUS-based pre-reconstruction, a novel setup was developed using a single reference sensor integrated into the template holder. The reproducibility and accuracy of the workflow using this latter setup was evaluated in phantom measurements.

**Results:**

The retrospective evaluation revealed that errors in active dwell positions of up to 10.2 mm caused absolute V_100%_ differences of up to 5.3 % of the target volume, and up to 24.4 %, 2.1 %, and 14.1 % of the prescribed dose for urethra D_0.1cm__3_, rectum D_2cm3_, and bladder D_1cm3_, respectively. In phantom measurements with our novel setup using reference sensor registration, EMT-based reconstruction demonstrated high reproducibility (0.5 mm) and agreement with TRUS-based reconstruction (0.7 mm), requiring a measurement time of < 23 s per implanted needle.

**Conclusion:**

The implementation of EMT-based reconstruction on clinical data shows clinically relevant impact on dose-volume metrics of TRUS-based implant reconstructions errors. Using the novel workflow and hardware, highly accurate and automated implant reconstruction is possible.

## Highlights

• Automated electromagnetic tracking enables accurate implant reconstruction.
• Electromagnetic tracking is integrated in transrectal ultrasound-guided prostate brachytherapy.
• Six-degree-of-freedom reference sensor allows easy coordinate system registration.
• Method has improved reconstruction reproducibility and reduces manual errors.

## Graphical abstract

**Figure:**
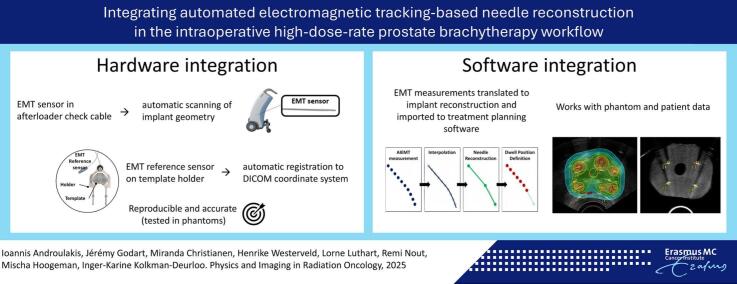

## 1 Introduction

High-dose-rate brachytherapy (HDR-BT) is an effective treatment for prostate cancer, either combined with external beam radiotherapy (EBRT) or as monotherapy [1], [2]. Transrectal ultrasound (TRUS) is the gold standard for guiding interstitial needle implantation in the operating room (OR). In case of intraoperative online treatment planning and delivery [2], [3] implant reconstruction is commonly performed using TRUS imaging [4], [5], [6]. However, identifying the correct position of the needles in TRUS images is difficult due to noise, artifacts, and shadowing effects [7], [8], [9]. Also, manual TRUS-based implant reconstruction can be a time-consuming and user-dependent process [10], [11], [12].

Electromagnetic tracking (EMT) has been proposed as an alternative method for implant reconstruction in TRUS-based HDR-BT settings [13]. EMT shows submillimeter accuracy in both laboratory and clinical settings [13], [14], [15], [16], [17], [18], [19], [20]. Moreover, in recent studies it has shown great potential as a tool for pretreatment verification of reconstruction accuracy in prostate BT patients [9], [20].

A comprehensive HDR-BT system that integrates EMT in a rigid implantation stylet was earlier developed and evaluated [12], [21], [22], [23], demonstrating a reduction of 10 min in implant reconstruction time by enabling a pre-reconstruction process that can be fine-tuned using TRUS images [12]. However, a limitation of this approach is that the resulting EMT-based pre-reconstruction does not fully represent the final implant geometry because needle tracking is performed during, instead of after, implantation, while the rigid stylet may deform the shape of the needle such that it is not representative of its position during irradiation. Consequently, manual correction based on TRUS images is still required.

An HDR-BT afterloader prototype with an integrated EMT sensor in the check cable, enabling it to follow the exact path of the radioactive source, has also been developed, and evaluated in clinical feasibility studies for breast, prostate, and cervical treatments to serve as a quality assurance tool [24], [25], [26]. Specifically, in TRUS-based prostate HDR-BT, a large number of reconstruction errors was previously identified when using this system [9].

It is hypothesized that performing implant reconstruction based on afterloader-integrated EMT measurements rather than on TRUS images might result in qualitatively better implant reconstruction and reduce treatment planning hands-on time in a procedure that can be labor- and time-intensive. Currently, software integration that enables the conversion of afterloader-integrated EMT measurements into implant reconstruction within the existing clinical treatment planning system is lacking. Moreover, with the current hardware, aligning EMT measurements with imaging coordinates (DICOM coordinate system) requires an existing reconstruction based on imaging information. The main aim of this study therefore was to integrate and validate an EMT-based automated implant reconstruction into the standard intraoperative HDR prostate workflow.

## 2 Materials and methods

We developed the necessary tools to enable EMT-based implant reconstruction in the treatment planning system. We retrospectively applied this workflow to prior EMT measurements and planning data. Furthermore, we developed and tested a method to register the EMT-based implant reconstruction to the TRUS-based anatomical delineations without relying on US-based (pre)reconstruction of the needles, simplifying the clinical workflow, by implementing a single six-degree-of-freedom (6DoF) reference sensor integrated into the implantation template holder, which registered the EMT measurements to the DICOM coordinate system.

### 2.1 EMT/TRUS based treatment planning hardware description

A Flexitron afterloader prototype (Elekta AB, Stockholm, Sweden), integrated with the Aurora EMT system (NDI, Waterloo, Canada), previously validated in [16], [17], [18], [19] was used. Details about prototype, hardware, and software setup can be found in the Supplementary material A. Online TRUS-based treatment planning was performed using the Oncentra Prostate v.4.2.3 (Elekta AB, Stockholm, Sweden) treatment planning system (TPS).

### 2.2 Needle reconstruction based on EMT measurements

A method was developed to generate the implant reconstruction based on EMT measurements and import it into the RTplan DICOM file using MATLAB 2021b (MathWorks, Natick, MA, USA). It was based on two inputs: raw EMT measurements and an RT-plan DICOM file.

**Fig. 1:**
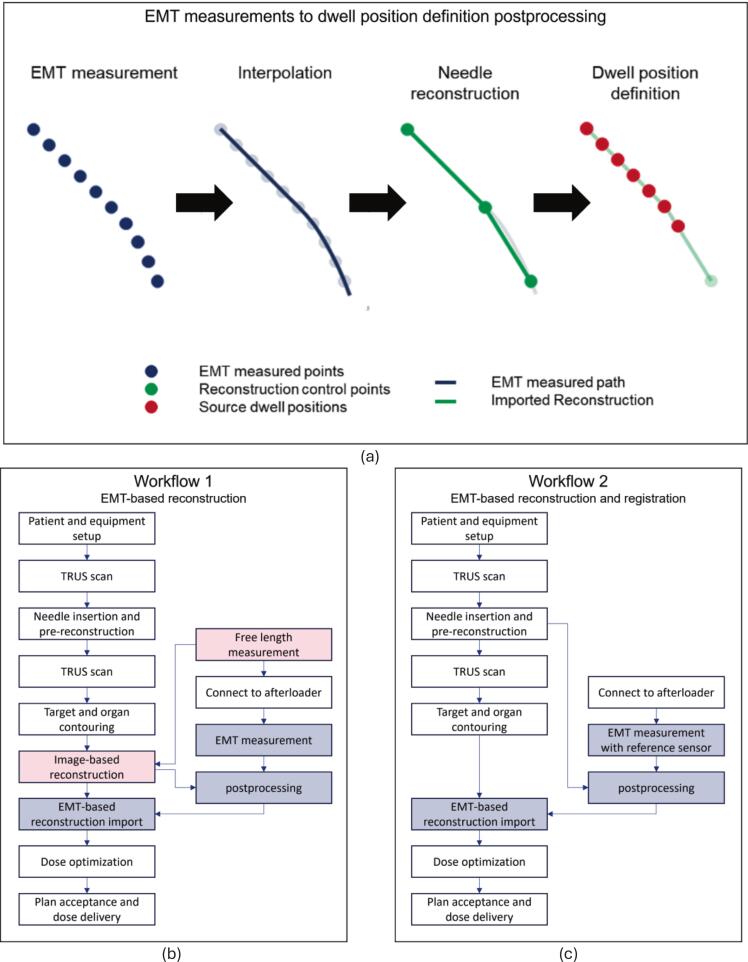
(a) The EMT-based reconstruction process. It is based on the import of EMT measurements aligned with the DICOM coordinate system. Then the EMT measurements are interpolated for each needle and on the interpolated spline needle reconstruction control points are defined. These needle control points are defining the implant reconstruction, where dwell positions can be derived from. (b) The ‘EMT-based reconstruction’ workflow using a TRUS-based (pre)reconstruction for registration of the EMT measurements to the DICOM coordinate system (Workflow 1). (c) The ‘EMT-based reconstruction and registration’ workflow using a 6DoF reference sensor for registration of the EMT measurements to the DICOM coordinate system (Workflow 2). Steps illustrated as white boxes are part of the current clinical workflow, blue boxes are added to enable EMT-based reconstruction, and red boxes can be omitted in Workflow 2. (For interpretation of the references to color in this figure legend, the reader is referred to the web version of this article.)

EMT measurements were conducted using a step-and-shoot protocol, measuring positions extending to the most distal dwell position of each implanted needle covering the total length. The raw EMT data were preprocessed and organized into a set of measurement points that corresponded to the sensor dwell positions of each needle, as described in previous studies [9], [18], [25]. Spline interpolation was applied between the measurement points of each needle to define the actual needle path. Based on the spline, the needle reconstruction and dwell positions were defined according to the TPS formalism. These were imported back in the TPS in the form of an adapted RT-plan DICOM file (Fig. 1. a). More details about the needle reconstruction method implementation can be found in Supplementary material B.

### 2.3 Registration to treatment plan coordinate system

The EMT measurement points need to be registered to the DICOM coordinate system of the RTplan in which anatomical delineations were defined. This could be done using two different registration methods, based on which two clinical workflows were proposed (Fig. 1.b-c). Workflow 1 used automated EMT measurements but required manual digitization for registration, whereas Workflow 2 achieved full automation by using a reference sensor for direct registration to the DICOM coordinate system. The first registration method, which was used in Workflow 1 and could be applied with the currently existing hardware, is described in Suplementary material C.

To automate and enable direct registration of EMT reconstructed needles to the DICOM coordinate system of the RTplan, a novel template holder prototype with an integrated Aurora 6DoF EMT reference sensor was designed for the Martinez Prostate Template (Elekta AB, Stockholm, Sweden). Direct registration was performed using the measured reference sensor position and orientation in the EMT coordinate system and the known position and orientation of the reference sensor relative to the DICOM coordinate system as described in Supplementary material D. With this registration method, the less labor-intensive Workflow 2 was enabled.

### 2.4 Retrospective implementation on clinical data

Workflow 1 was retrospectively implemented on data of fourteen prostate cancer patients, treated using intraoperative HDR BT using TRUS-based treatment planning in which EMT measurements were performed as part of a study (MEC-2018–1559, date of approval 13–11-2018). Further details of the treatment and measurement protocol can be found in [9], [25]. EMT-based treatment plans with the same dwell times as in the clinical treatment plan were created, reflecting the delivered dose distribution to patients, as high EMT accuracy in the intraoperative setting was demonstrated [25], supporting its use as a reliable reference. The target and organ-at-risk dose-volume histogram parameters of the original TRUS-based treatment plan were compared with those of the EMT-based treatment plan. Thus, the impact on dose-volume metrics of reconstruction errors previously identified and validated using EMT measurements and TRUS image review [9], which could have been avoided by using EMT-based reconstruction instead of TRUS-based reconstruction, was evaluated.

### 2.5 Implementation of the novel registration method and evaluation in phantom

Workflow 2 was evaluated using a phantom with four inserted needles in the brachytherapy treatment room (Fig. S1). Needles were positioned in a way that ensured high reliability of the TRUS-based implant reconstruction, so that it could serve as ground truth for comparison to the EMT-based reconstruction of Workflow 2. Details about the setup can be found in Supplementary material E.

EMT measurements were conducted at 11 positions spaced 5 mm apart, covering a total length of 50 mm. Reconstruction reproducibility was evaluated by repeating the entire reconstruction process four times, including the reconnection of the afterloader to the implant and repositioning of the field generator, but without moving the template and stepper to avoid displacing the implanted needles. The positional deviation between the dwell positions resulting from repeated reconstructions was calculated. Reproducibility was defined as the statistical uncertainty of the reconstructions [27], expressed by two standard deviations (SD) (k = 2) following a 95 % confidence interval.

The reconstruction accuracy was calculated as the Euclidean distance (ED) between the EMT-based and reference TRUS-based reconstructions. TRUS-based reconstruction was performed according to the original clinical protocol [9]. This accuracy is dependent on the ability of the afterloader-integrated EMT sensor to measure the correct implant geometry, as well as on the ability of the template-integrated reference sensor method to correctly register the EMT measurements to the imaging coordinate system. Offline TRUS-based reconstruction was performed by three experienced radiotherapy technologists (RTTs). The average of the three reconstructions was used as the reference reconstruction, and the deviation between the three reconstructions was used to define interobserver variation. The imaging geometric uncertainty was < 2 mm, according to the TRUS system commissioning results [3]. The needle depth uncertainty was regarded < 1 mm, as it was defined using free-length measurements with a ruler with millimeter resolution [4].

## 3 Results

**Fig. 2:**
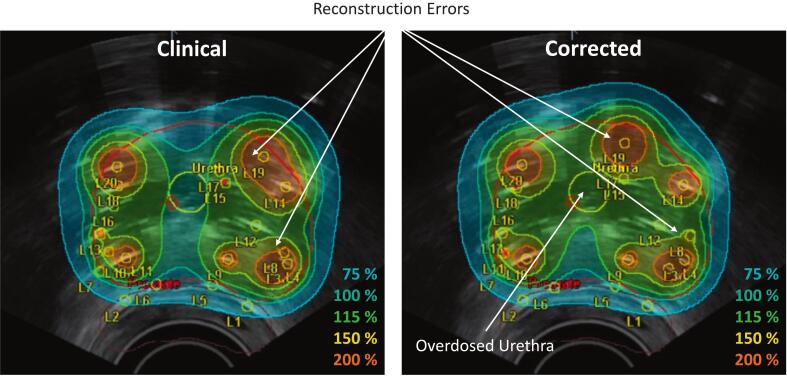
Retrospective implementation of the EMT-based implant reconstruction and comparison to the clinically applied TRUS-based reconstruction: Example case of patient with patient ID 12, where it is evident that erroneous needle reconstruction for needles L8 and L19 have caused serious deformation of the dose distribution in the clinical plan. The actually delivered dose resulted in an overdosed urethra when looking at the 115% isodose curve.

**Fig. 3:**
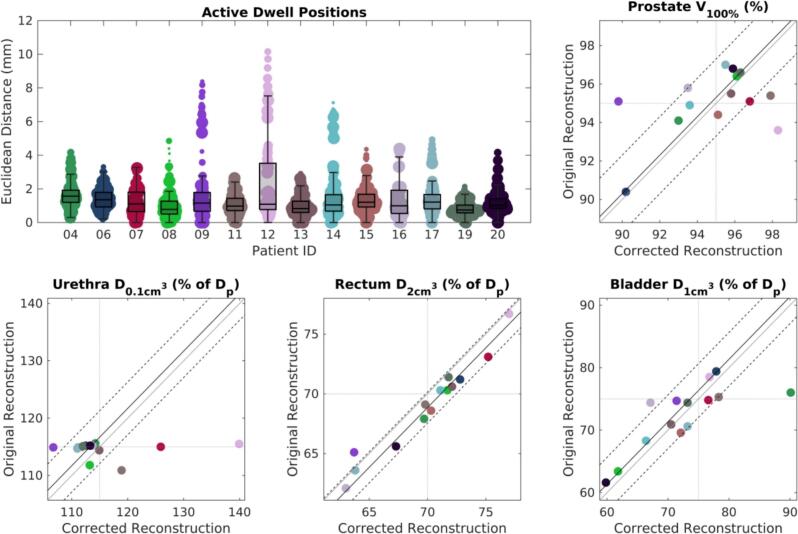
Summarized results for Euclidean distance between active dwell positions in the clinical TRUS-based reconstruction and the EMT-based reconstruction, and differences in Prostate V_100%_, Urethra D_0.1cm3_, Rectum D_2cm3_, and Bladder D_1cm3_. Results are color-coded per patient, and in the Euclidean distance plot, point size corresponds to dwell time at that position. In the dose-volume metrics plots: the gray diagonal line is the equality axis; the continuous and dashed black diagonal lines are the median and interquartile lines; the dotted gray horizontal and vertical lines are the dose objectives or constraints in the treatment plan. Boxplots display the median (line), the 25th and 75th percentiles (box edges), and whiskers extending to the most extreme values within 1.5 times the interquartile range.

For the retrospective implementation of EMT-based needle reconstruction on clinical data, comparing dose distributions calculated using clinical TRUS-based and EMT-based implant reconstructions showed that deviations such as two needles with large deviations could lead to significant changes in the dose distribution, including urethral overdosing (Fig. 2). For all the patients the median (range) ED between the original and corrected active dwell positions was 1.1 (0.0 – 10.2) mm (Fig. 3). In terms of target coverage, this resulted in a median (range) V_100%_ difference of −0.3 (−5.3 – 4.7) % of the target volume (Fig. 3). In terms of organs at risk, the median (range) differences for urethra D_0.1cm_^3^ and bladder D_1cm_^3^ were −1.7 (−8.2 – 24.4) % and −1.4 (−7.3 – 14.1) %, respectively, though lower for rectum D_2cm_^3^, that is 1.15 (−1.40 – 2.10) % (Fig. 3). Differences for prostate V_100%_, urethra D_0.1cm_^3^, and bladder D_1cm_^3^ were not statistically significant (Wilcoxon sign-rank, p = 0.58, p = 0.51, and p = 0.92), whereas rectum D_2cm_^3^ demonstrated a significant reduction with EMT-based reconstruction compared to TRUS-based reconstruction (Wilcoxon sign-rank, p = 0.002).

**Fig. 4:**
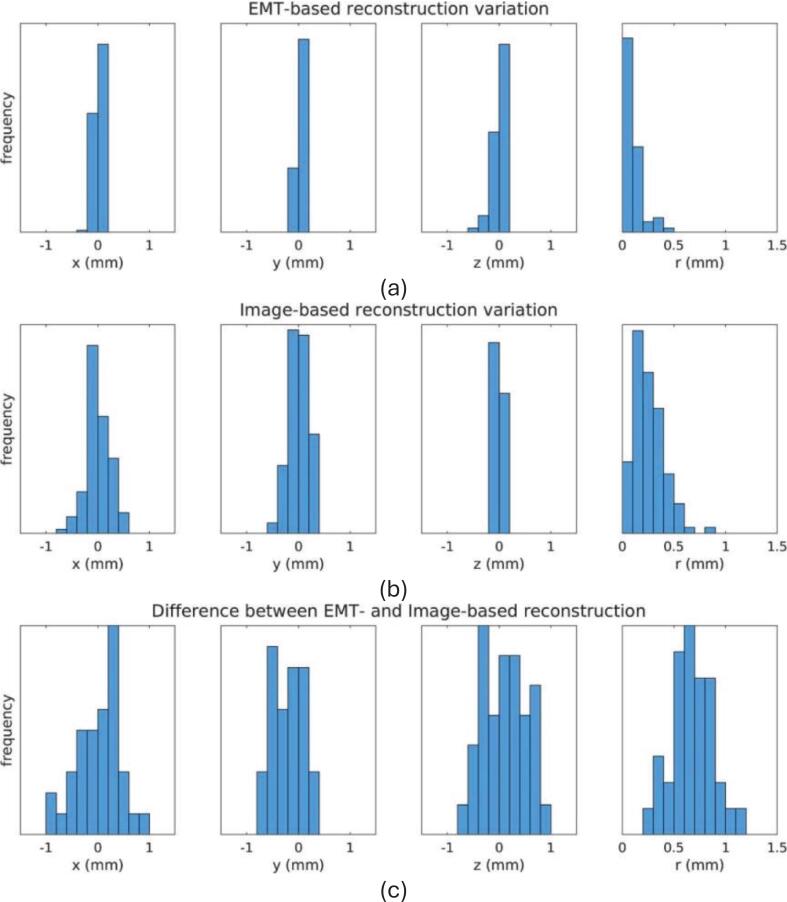
Histograms showing the (a) EMT-based reconstruction variation, (b) image (TRUS)-based reconstruction variation, and (c) difference between EMT- and TRUS-based reconstructions in the x (medio-lateral), y (anterior-posterior), z (superior-inferior) axes, and r (Euclidean distance) of the DICOM coordinate system, as recorded during phantom experiments.

For the phantom evaluation of workflow 2 with the novel registration method, EMT measurements for all four needles were performed in < 90 s, resulting in a required time per needle of < 23 s. Processing and subsequent implant reconstruction export were performed in < 30 s. The EMT-based reconstruction reproducibility (k = 2) in the target region was 0.47 mm. Looking at each Cartesian axis, the reproducibility was 0.11 mm, 0.13 mm, and 0.19 mm in the x (medio-lateral), y (anterior-posterior), and z (superior-inferior) axes of the imaging coordinates, respectively (Fig. 4.a). TRUS-based reconstruction had a reproducibility (k = 2) of 1.04 mm. Considering each Cartesian axis independently, the reproducibility was 0.45 mm, 0.41 mm, and 0.09 mm in the x, y, and z axes of the imaging coordinates, respectively. (Fig. 4.b). Significantly better x-axis, y-axis, and radial reproducibility for the EMT-based reconstruction were observed (Student’s *t*-test, p-value < 0.001). The mean (±2SD) agreement between the two reconstruction methods, expressed by the Euclidean distance of the corresponding dwell positions within the prostate region, was 0.67 (±0.37) mm. Mean (±2SD) differences were 0.04 (±0.84) mm, −0.19 (±0.61) mm, and 0.13 (±0.83) mm for the x, y, and z axes of the imaging coordinates, respectively (Fig. 4.c). Despite the magnitude being small, bias between EMT and TRUS-based reconstructions was significant on the y-axis (Student’s *t*-test, p = <0.001).

## 4 Discussion

We developed a method to perform automated implant reconstruction based on electromagnetic tracking measurements in intraoperative prostate HDR BT. We successfully applied the method retrospectively to clinical cases with previously identified reconstruction errors and evaluated the differences on dose-volume metrics. We also developed and evaluated a novel setup by registering the EMT-based reconstruction to the DICOM coordinate system using a single reference sensor integrated into the template holder. Thus, the EMT-based reconstruction method can be registered directly and is less labor-intensive, as there is no longer a need for manual implant reconstruction or physical free length measurements. We successfully implemented this in phantoms and demonstrated highly reproducible reconstruction geometries that were in good agreement with the TRUS-based reconstructions.

The retrospective implementation on clinical data showed large differences on dose-volume metrics between the original TRUS-based implant reconstructions and the corrected EMT-based reconstructions in case the active dwell positions were affected by reconstruction errors. We observed differences of up to 5.3 % in target coverage (V_100%_) and high differences of up to 24.4 % and 14.1 % in urethral (D_0.1cm_^3^) and bladder (D_1cm_^3^) exposures, respectively. Maximum differences were of lower magnitude for rectum (D_2cm_^3^), likely due to the better image quality near the TRUS probe leading to less large errors. However, over all evaluated patients there was a small but significant difference, indicating a possible systematic change in distance between needles and organ. A limitation of this single institution evaluation is that these results might depend on the planning technique of the department. Nevertheless, these results are in general consistent with the findings of Deufel et al. who used EMT measurements in a similar setting to evaluate the clinically delivered treatment [20]. A difference with Deufel et al., however, is that there were no implants with very large errors in their study, leading to also lower differences in dose-volume metrics compared to the current study. Implementing EMT-based reconstruction in clinical practice could result in less geographical misses of the prostate and a reduced risk of unintended radiation exposure to surrounding organs, such as the urethra and bladder. This may potentially enhance treatment efficacy and reduce side effects.

In the phantom setup EMT-based implant reconstruction exhibited superior reproducibility compared to the TRUS-based approach, even with optimal conditions being established for TRUS-based reconstruction that effectively minimize distortions and artifacts. In clinical practice, TRUS-based reconstruction reproducibility is expected to be lower owing to increased shadowing, image distortions, and other uncertainties, which can result in larger inter-observer variability [11]. Considering the lower reproducibility and the < 2 mm geometric uncertainty of the TRUS imaging system, the agreement between TRUS-based and EMT-based reconstructions is likely to be influenced more by the TRUS reconstruction uncertainty than the EMT reconstruction uncertainty. Hence, it can be concluded that although accuracy in this study is defined as the agreement between the TRUS-based and EMT-based reconstructions (0.67 mm), the actual EMT-based reconstruction accuracy is likely below that value.

In terms of accuracy, Bharat et al. found that an in-plane accuracy of less than 0.5 mm and a craniocaudal accuracy of less than 1 mm could be achieved with manual EMT measurements in a similar prostate phantom setup [13]. This aligns with the TRUS-EMT agreement observed in our study. Additionally, Beaulieu et al. utilized the EMT implantation needle in their evaluated system and reported an agreement between the TRUS image-annotated and EMT-measured needle tips of less than 1 mm [21]. Assuming that the needle tip location agreement in their study was analogous to the dwell position location agreement in our study, we can conclude that both systems demonstrated similar TRUS-EMT agreement. The differences between TRUS reconstructed and EMT reconstructed needles, will however remain dependent on the TRUS equipment used, patient anatomy and the experience of the user reconstructing the needles on the TRUS image. Therefore, more investigations in a multicenter setting would be beneficial.

When considering time efficiency, Poulin et al. reported that manual catheter reconstruction measurements using a rigid stylet could be completed in less than 3 min for a typical 17-needle implant [28]. Our proposed automatic reconstruction method requires approximately 6.5 min (23 s per needle) to perform the measurements. The entire EMT-based needle reconstruction process takes less than 10 min. Although the proposed protocol is slower than that proposed by Poulin et al., it is automated and integrated in the afterloader, making implementation simpler. One of the most important advantages is the automatic channel detection for each needle, due to the direct link between the afterloader channel and the position of the detected needle within the patient’s anatomy. Another advantage is the inherent ability of the system to detect the dwell position location in addition to the needle location. In addition, the proposed integration with the current clinical TPS leads to a smooth clinical workflow. Nevertheless, depending on the anticipated limited curvature of the needles, the number of measured positions may be reduced, or continuous motion measurement protocols may be used, as investigated by Tho et al. [17], which may result in a shorter measurement time.

In their study of clinical data, Lavallee et al. utilized manual EMT measurements using a rigid implantation stylet and found that in 38 % of cases, automatic reconstruction using EMT required manual corrections [12]. With the current setup, no such corrections are necessary because the EMT measurements are conducted with a source-like sensor that does not disturb the implant geometry by straightening the needle. Furthermore, the integration of the afterloader with the EMT as a reconstruction tool inherently eliminates user-related errors such as needle swaps or incorrect needle assignments. Additionally, with the proposed workflow (Workflow 2), free length measurement will serve primarily to verify the accuracy of implant reconstruction results, but can be omitted.

Our findings suggest that integrating EMT-based reconstruction into clinical practice can enhance the quality of brachytherapy for prostate cancer. In addition to providing more accurate implant reconstructions, this technology has the potential to reduce the workload and time associated with treatment preparation and to minimize user-dependent errors. Therefore, it would be beneficial if the EMT-enabled afterloader became commercially available for clinical use. To implement the proposed in-house developed workflow in clinical practice, the software and hardware add-ons need to be tested and approved for clinical use adhering to Article 5(5) of the European Medical Device Regulation 2017/745.

Other limitations should be adressed answered in future work. For the reference sensor holder, positional accuracy over time due to aging of the holder should be investigated in more detail, and a calibration protocol should be proposed accommodating possible deformation over time. Moreover, future prospective clinical trials are needed to capture practical issues possibly caused by anatomical variability.

In conclusion, in this study we achieved integration of EMT-based automated implant reconstruction into the intraoperative HDR prostate brachytherapy workflow. The developed EMT-based reconstruction and registration workflow (Workflow 2) demonstrated superior reproducibility and good agreement with the currently implemented TRUS-based reconstruction method under optimal conditions. By enabling full automation using a reference sensor, its integration into clinical practice promises to enhance treatment precision, reduce procedure time, and minimize user-dependent errors. EMT-based reconstruction was shown to reduce errors in dose-volume metrics, such as those in the evaluated dataset.

## Funding statement

The study was supported by Elekta AB, Stockholm, Sweden.

## CRediT authorship contribution statement

**Ioannis Androulakis:** Conceptualization, Methodology, Data curation, Software, Validation, Formal analysis, Investigation, Data curation, Writing – original draft, Visualization, Project administration. **Jérémy Godart:** Conceptualization, Methodology, Resources, Writing – review & editing, Supervision. **Miranda E.M.C. Christianen:** Conceptualization, Methodology, Resources, Writing – review & editing, Supervision. **Henrike Westerveld:** Conceptualization, Methodology, Resources, Writing – review & editing, Supervision. **Lorne Luthart:** Conceptualization, Methodology, Data curation, Validation, Writing – review & editing. **Remi A. Nout:** Conceptualization, Methodology, Resources, Writing – review & editing, Supervision. **Mischa S. Hoogeman:** Conceptualization, Methodology, Resources, Writing – review & editing, Supervision. **Inger-Karine K. Kolkman-Deurloo:** Conceptualization, Methodology, Resources, Writing – review & editing, Supervision, Project administration, Funding acquisition.

## Declaration of competing interest

The authors declare the following financial interests/personal relationships which may be considered as potential competing interests: This research was funded by Elekta AB, Stockholm, Sweden. The authors received hardware and hardware support from Elekta AB, Stockholm, Sweden.
