# Supplementary material for: Integrating automated electromagnetic tracking-based needle reconstruction in the intraoperative high-dose-rate prostate brachytherapy workflow
Source: Phys Imaging Radiat Oncol. 2025 Dec 3;36:100884. doi: 10.1016/j.phro.2025.100884 (PMC12722971; doi:10.1016/j.phro.2025.100884)
Supplement: Supplementary Data 1 [file mmc1.pdf]

Supplementary materials to the manuscript:

Integrating automated electromagnetic tracking-based needle reconstruction in the intraoperative high-dose-rate prostate brachytherapy workflow

Ioannis Androulakis[1,*], Jérémy Godart[1,2], Miranda E.M.C. Christianen[1], Henrike Westerveld[1], Lorne Luthart[1], Remi A. Nout[1], Mischa S. Hoogeman[1,2], Inger-Karine K. Kolkman-Deurloo[1]

[1] Department of Radiotherapy, Erasmus MC Cancer Institute, University Medical Center, Rotterdam, The Netherlands

[2] Department of Medical Physics and Informatics, HollandPTC, Delft, The Netherlands

*Corresponding Author: i.androulakis@erasmusmc.nl

Supplementary material A – Setup description

The afterloader prototype features a five-degree-of-freedom (5DoF) 5.9 mm length custom-made EMT sensor encapsulated in the tip of the check cable wire that can be navigated through the implanted plastic needles (Proguide; Elekta AB, Stockholm, Sweden) according to predefined dwell positions. When combined with a Planar 20-20 electromagnetic field generator (FG) (NDI, Waterloo, Canada) and a processing unit, the location of the sensor can be accurately determined with respect to the field generator. In an earlier evaluation we found that the EMT system performed equally well in the proximity of the stepper and TRUS probe, as in a disturbance-free environment [1]. Quality assurance of the EMT system followed a published protocol [1]. In addition to the prototype afterloader, an OncoSelect mechanical stepper (Elekta AB, Stockholm, Sweden), along with a bkSpecto Ultrasound System and an E14CL4b Endocavity Biplane Transducer (BK Medical, Burlington, MA, USA), were used for image guidance during implantation and treatment planning. The ultrasound system was commissioned in accordance with the GEC/ESTRO recommendations [1].

Treatment planning was based on three-dimensional (3D) TRUS volumes acquired by automated rotation of the TRUS transducer on the stepper and resampling the acquired sagittal images from the scanned volume to a 3D grid with a voxel resolution of 0.2 mm in all three dimensions.

During the EMT measurements, the field generator was positioned above the patient, keeping a distance of 5 cm from the skin, aligning as much as possible the center of the measurement volume with the center of the target area of the patient.

Supplementary material B – EMT-based needle reconstruction details

EMT-based needle reconstruction was based on two inputs: raw EMT measurements and an RT-plan DICOM file. The patient-specific RT plan DICOM file needed to already contain the needle definitions. This could be either a preplanned needle definition with default position values (in the Virtual Plan mode of the TPS with needles fixed to template grid coordinates) or TRUS-based (pre)reconstructed needles (in the Live Plan mode of the TPS).

EMT measurements were conducted using a step-and-shoot protocol, measuring positions extending to the most distal dwell position of each implanted needle covering the total length.

The raw EMT data were preprocessed and organized into a set of measurement points that corresponded to the sensor dwell positions of each needle, as described in previous studies [3, 4, 5]. Spline interpolation was applied between the measurement points of each needle to define the actual needle path. Then, for each needle defined in the RTplan, a set of control points was automatically generated (default position values in Virtual Plan mode) or adapted by the user (reconstruction position values in Live Plan mode) and stored. Needle reconstruction was defined as the linear interpolation between the coordinates of these control points, with the first control point corresponding to the most distal dwell position. The existing control points in RTplan were modified according to the spline defined by the EMT measurement points. An updated RTplan file incorporating the EMT-based needle reconstruction was created (Fig. 1. a), which can be imported directly into the TPS. The source dwell positions along each needle were defined and adapted based on the RTplan needle reconstruction in the clinical software. We implemented the same number of control points as originally present in the RTplan, meaning that the needle path was modelled with a lower resolution than the EMT points along each needle would allow. This was considered sufficient for this type of needles.

Supplementary material C – Image-based EMT-to-DICOM registration method

The image-based registration method required the presence of a TRUS-based (pre)reconstruction in the RTplan. In this method, the measurement points were registered to the corresponding pre-reconstructed dwell positions in the RTplan using coherent point drift (with an outlier ratio of 10%) as described previously [3, 5].

Supplementary material D – Reference sensor-based EMT-to-DICOM registration method

The EMT-to-DICOM transform was calculated as:

$$^{DICOM}T_{EMT} = {}^{DICOM}T_{TMP} \cdot {}^{TMP}T_{REF} \cdot ({}^{EMT}T_{REF})^{-1} \quad (1)$$

where $^{DICOM}T_{TMP}$ is the transform from the template to the DICOM coordinate system, which is constant except for the longitudinal offset along the stepper; $^{TMP}T_{REF}$ is the known fixed position and orientation of the reference sensor relative to the template based on physical dimensions and mounting configuration; and $^{EMT}T_{REF}$ is the measured 6DoF position and orientation of the reference sensor in the EMT coordinate system. No phantom or image-based calibration was performed for the definition of $^{TMP}T_{REF}$; however, the modeled reference sensor position was visually validated by inspecting the alignment of needle measurements to the template coordinates at the template plane.

Supplementary material E – Phantom setup

Workflow 2 was evaluated using a tissue-equivalent ultrasound prostate phantom (Model 053S, CIRS INC, Norfolk, VA, USA) in a brachytherapy treatment room (Figure S1.b). Four 240 mm needles were inserted through the Martinez template in a rectangular shape (at positions E2, C2, E3.5, and C3.5), ensuring that the anterior needles were not shadowed by the posterior needles and that the implantation geometry was well identifiable on TRUS images (Figure S1.c). This was

done to ensure high reliability of the TRUS-based implant reconstruction serving as ground truth for comparison to the EMT-based reconstruction of Workflow 2.

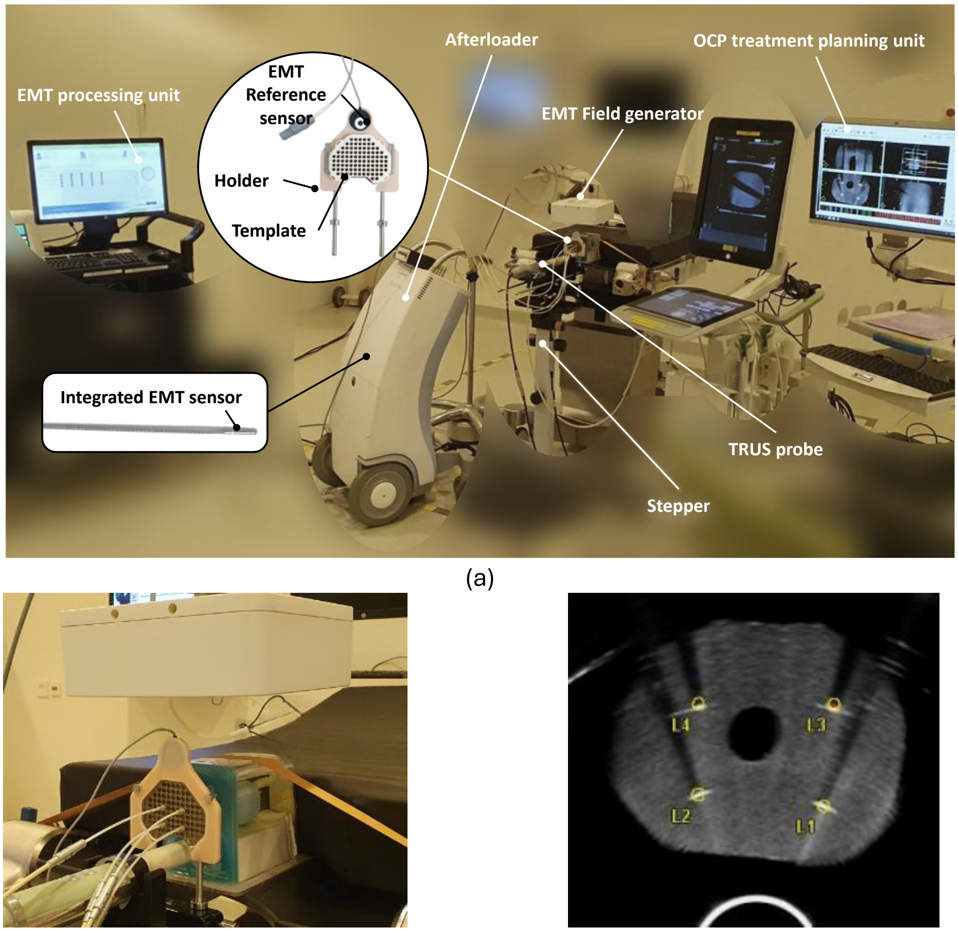

Figure S1 – *(a) the proposed OCP-EMT setup in the brachytherapy room consisting of the afterloader with integrated EMT sensor and EMT field generator and EMT processing unit, the TRUS probe and imaging unit, the OCP treatment planning unit, the stepper and template with integrated EMT sensor are visible. (b) The phantom setup used to evaluate the EMT-based implant reconstruction workflow. (c) TRUS imaging reference transversal plane view in the treatment planning software, showing the four implanted needles in the prostate phantom as they were placed in the phantom experiments. The yellow circles show the manual TRUS-based implant reconstruction.*

References

[1] Dürrbeck C, Gomez-Sarmiento IN, Androulakis I, Sauer BC, Kolkman-Deurloo IK, Bert C, Beaulieu L. A comprehensive quality assurance protocol for electromagnetic tracking in brachytherapy. Med Phys. 2024;51:3184-94. https://doi.org/10.1002/mp.17017

[2] Siebert FA, Kirisits C, Hellebust TP, Baltas D, Verhaegen F, Camps S, Pieters B, Kovács G, Thomadsen B. GEC-ESTRO/ACROP recommendations for quality assurance of ultrasound imaging in brachytherapy. Radiother Oncol. 2020;148:51-6. https://doi.org/10.1016/j.radonc.2020.02.024

[3] Androulakis I, Godart J, Luthart L, Christianen MEMC, Westerveld H, Nout RA, Hoogeman M, Kolkman-Deurloo IK. Reconstruction errors in clinical intraoperative TRUS–based prostate HDR-BT detected using electromagnetic tracking. Brachytherapy. 2025;24:177-85. https://doi.org/ 10.1016/j.brachy.2024.11.004

[4] van Heerden L, Schiphof-Godart J, Christianen M, Mens J-W, Franckena M, Maenhout M, Hoogeman M, Kolkman-Deurloo IK. Accuracy of dwell position detection with a combined electromagnetic tracking brachytherapy system for treatment verification in pelvic brachytherapy. Radiother Oncol. 2021;154:249-54. https://doi.org/10.1016/j.radonc.2020.09.061

[5] Androulakis I, Schiphof-Godart J, van Heerden LE, Luthart L, Rijnsdorp R, Hoogeman MS, Westerveld H, Christianen ME, Mens JW, van Paassen R, Negenman EM, Nout RA, Kolkman-Deurloo IK. Assessment of integrated electromagnetic tracking for dwell position monitoring in a clinical HDR brachytherapy setting for prostate cancer. Radiother Oncol. 2024:110501. https://doi.org/10.1016/j.radonc.2024.110501
